# Direct and Convenient Mass Spectrometry Sampling with Ambient Flame Ionization

**DOI:** 10.1038/srep16893

**Published:** 2015-11-19

**Authors:** Xiao-Pan Liu, Hao-Yang Wang, Jun-Ting Zhang, Meng-Xi Wu, Wan-Shu Qi, Hui Zhu, Yin-Long Guo

**Affiliations:** 1 State Key Laboratory of Organmetallic Chemistry and National Center for Organic Mass Spectrometry in Shanghai, Shanghai Institute of Organic Chemistry, Chinese Academy of Sciences, 345 Lingling Road, Shanghai, 200032 (China)

## Abstract

Recent innovations in ambient ionization technology for the direct analysis of various samples in their native environment facilitate the development and applications of mass spectrometry in natural science. Presented here is a novel, convenient and flame-based ambient ionization method for mass spectrometric analysis of organic compounds, termed as the ambient flame ionization (AFI) ion source. The key features of AFI ion source were no requirement of (high) voltages, laser beams and spray gases, but just using small size of *n*-butane flame (height approximately 1 cm, about 500 ^o^C) to accomplish the rapid desorption and ionization for direct analysis of gaseous-, liquid- and solid-phase organic compounds, as well as real-world samples. This method has high sensitivity with a limit of detection of 1 picogram for propyphenazone, which allows consuming trace amount of samples. Compared to previous ionization methods, this ion source device is extremely simple, maintain-free, low-cost, user–friendly so that even an ordinary lighter (with *n*-butane as fuel) can achieve efficient ionization. A new orientation to mass spectrometry ion source exploitation might emerge from such a convenient, easy and inexpensive AFI ion source.

The features of high specificity, high speed and high sensitivity make mass spectrometry a powerful and versatile tool in the field of the analytical science. To meet the ever-increasing demand of nature science, it is urgent to develop new and effective ionization technologies of mass spectrometry to produce target ions. Traditional soft ionization methods, e.g., electrospray ionization (ESI)^1^,^2^,^3^, matrix-assisted laser desorption ionization (MALDI)^4^,^5^,^6^, have largely expanded the scopes of MS ionization, capable to handle various molecules with wide range of polarities and molecular weights, for example proteins, bacteria and metabolites. However, they require intricate sample pretreatments and complex devices, which render disturbance of analyte environment, requirement of multi-parameter adjustment and specialized training. The inventions of desorption electrospray ionization (DESI)^7^,^8^,^9^ and direct analysis in real time (DART)^10^,^11^ have triggered overwhelming development breakthrough of mass spectrometry. Namely, a new family of ionization methods occurs, known as ambient mass spectrometry without tedious sample preparation and operation in sample natural environment^12^,^13^,^14^,^15^,^16^,^17^,^18^,^19^,^20^. The past ten years witnessed the rapid development of ambient ionization methods which are becoming increasingly prosperous. Although ambient ionization technologies have simplified analytical procedure and improved ionization capabilities, some are still inconvenient given that the auxiliary gases, solvents, laser beams, electrical power consumption, etc. are required for operation. Several of these ambient ionization methods include matrix-assisted laser desorption electrospray ionization (MALDESI)^21^, laser ablation metastable-induced chemical ionization(LAMICI)^22^, desorption electrospray/metastable-induced ionization(DEMI)^23^, plasma assisted multiwavelength laser desorption ionization(PAMLDI)^24^, and ambient pressure thermal desorption ionization (APTDI)^25^,^26^, etc^27^,^28^,^29^. For further enlarging the application scopes of ambient ionization technologies, there is still a great necessity to engage in developing a more convenient and simpler ionization method to make whenever and wherever *in-situ* MS analysis feasible.

As we all know, the use of fire has paramount significance in the human civilization history and the applications of flame in the field of analytical science also have abundant history. The widely used flame-based analytical technologies need high temperature (usually up to thousands of degrees) to provide enough energy to atomize the analytes for further spectroscopy analyses, such as flame atomic absorption spectroscopy (up to 2000 ^o^C)^30^, flame atomic emission spectroscopy^31^, flame atomic fluorescence spectroscopy^32^, and inductively coupled plasma mass spectrometry (up to 4000 ^o^C)^33^. These technologies have been widely applied to constant and trace element analysis in the fields of metallurgy, geology, mining, medicine, sanitation, and food. Flame-based chromatographic detectors (e.g. flame ionization detector^34^, nitrogen phosphorus detection^35^, flame photometric detector^36^) used in gas chromatography (GC), high performance liquid performance (HPLC) and thin layer chromatography (TLC) have been extensively reported. These detectors take advantage of flame to combust organic compounds separated from GC, HPLC, TLC and detect electrical signal stemming from the transformation of ion currents. Some papers reported flame-based ionization sources, for example air-acetylene flame^37^ and air-hydrogen flame^38^, which also reached to thousands of temperature and were used for the determination of isotope ratio of element.

Inspired by the idea of the ion generation processes from the flame, herein we describe the development and application of a highly convenient ambient ionization technology, ambient flame ionization (AFI) ion source. The flame temperature of AFI-MS is comparatively lower at about 500 ^o^C (when *n*-butane was used as fuel and with low flow rate about 15 ml/min) and the experimental results showed that AFI-MS was an ideal soft ionization technology for analysis of organic compounds (usually generating quasi-molecular ion) by fast touching the samples with outer flame without obvious signals caused by degradation, oxidation and pyrolysis. AFI only requires ambient flame, that is, operation in a laser-, high voltage-, spray gases -free condition. The capability of AFI was verified by analyzing organic compounds and various real-world samples without intricate pretreatment prior to analysis. The applicability of AFI in analytical science was demonstrated by the direct and convenient analysis of real-world samples with high sensitivity, for example, pharmaceutical tablets, xenobiotics in fruit peels and vegetable surfaces, and even pork fat.

## Results

### AFI instrumentation

**Figure 1:**
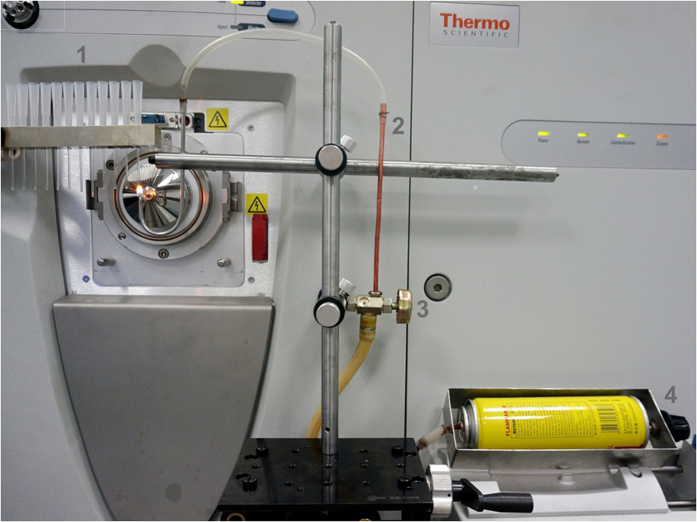
The photograph of ambient flame ionization mass spectrometry (AFI-MS) apparatus for direct analysis of the sample. 1. sample rods, 2. butane transferring pipe, 3. flow micro-adjust valve, 4. pressure controlling valve. The analytes could be desorbed and ionized rapidly in the outer flame region, and the ions generated by AFI could be transferred and detected by the mass spectrometry. The optimal distance between the centre of flame and the inlet to mass spectrometer is 10 mm. The ideal size of butane flame (height about 1 cm) is controlled by pressure controlling valve and exactly adjusted by flow micro-adjusting valve.

Figure 1 shows the photograph of AFI-MS device. In this device, the size of flame is controlled by pressure controlling valve and exactly adjusted by the flow micro-adjusting valve. An array of sample rods are prone to performing sequence sample analysis. In the AFI, the process of fuel combustion (here is *n-*butane) produces abundant energies which are released and translated to kinetic energy. The high-speed collisions of fast-moving reactive species in the *n-*butane flame go through various chemical ionization reactions. The reactive species in the flame of alkane were reported, such as: CHO^+^ and H_3_O^+^^39^,^40^,^41^,^42^,^43^. The ion-molecule reactions of these reactive species in the *n-*butane flame with analytes were important for the ionization of AFI-MS. The experimental results showed that the major ionization mode of AFI-MS is protonation of various organic compounds. The ions in the gas phase formed in the outer flame region could be easily transferred into MS entrance caused by the vacuum of mass spectrometer (Supplementary Fig. S1). Therefore, the distance of the flame to the MS entrance is important for the sensitivity of the AFI-MS method. The result demonstrated that the optimal distance between the center of flame and the inlet to mass spectrometer was 1 cm (Supplementary Fig. S2a).

Flames of different fuels were compared, such as alkane, alcohol, ester and ketone (Supplementary Fig. S2b). The results demonstrated that the optimal fuel was *n-*butane. Compared with *n-*butane, they give much poor performance in the same experiment protocol. What is more, the gas nature of *n-*butane is easy to control by changing flow rate; however, the generation of flame about liquid fuels, such as alcohol, ethyl acetate and acetone have to use cotton thread and the flame size is not easy to control. Considering the most important ionization is protonation in AFI-MS analysis, it was proposed that the much lower proton affinity of *n-*butane (the data of proton affinity of *n-*butane is not available, data of *iso*-butane is referred)^44^ than the analytes might be a critical reason for the higher ionization performance of *n-*butane. In the process of analysis, different introduction points of flame were compared (supplementary Table S1 and Supplementary Fig. S1). The result showed that samples directly subjected to the outer flame were optimal in view of relative standard deviation and signal/noise. It is possible that the combustion of outer flame generates high temperature and more active species, which was beneficial for desorption and ionization of samples (Supplementary Fig. S2). The nature of flame can be controlled by changing the parameters of combustion, such as fuel flow rate and flame size (supplementary Table S2). In the AFI-MS analysis, the flow of butane is low (usually 15 mL/min) and no assisting air or oxygen flow is applied. Thus the flame temperature in the AFI-MS experiments usually remains at about 500 ^o^C and is ideal for fast analysis of organic compounds, which is able to accomplish the rapid desorption and ionization of analyte and usually does not cause the obvious thermal degradation and oxidation of the analytes.

What is more, the device of AFI ion source is extremely simple and portable which only calls for ordinary flame to perform ionization without assistances of (high) voltages, laser beams, and spray gases. Rather amazingly, the lighter (with *n*-butane as fuel) was also able to accomplish ionization (Supplementary Fig. S3). As an ionization source, the lighter is easy to carry and operate, lightweight, universally available and convenient, especially combining with portable mass spectrometry^45^,^46^,^47^ for *in-situ* and *in-field* analysis.

### Analysis of trace vapor molecules

**Figure 2:**
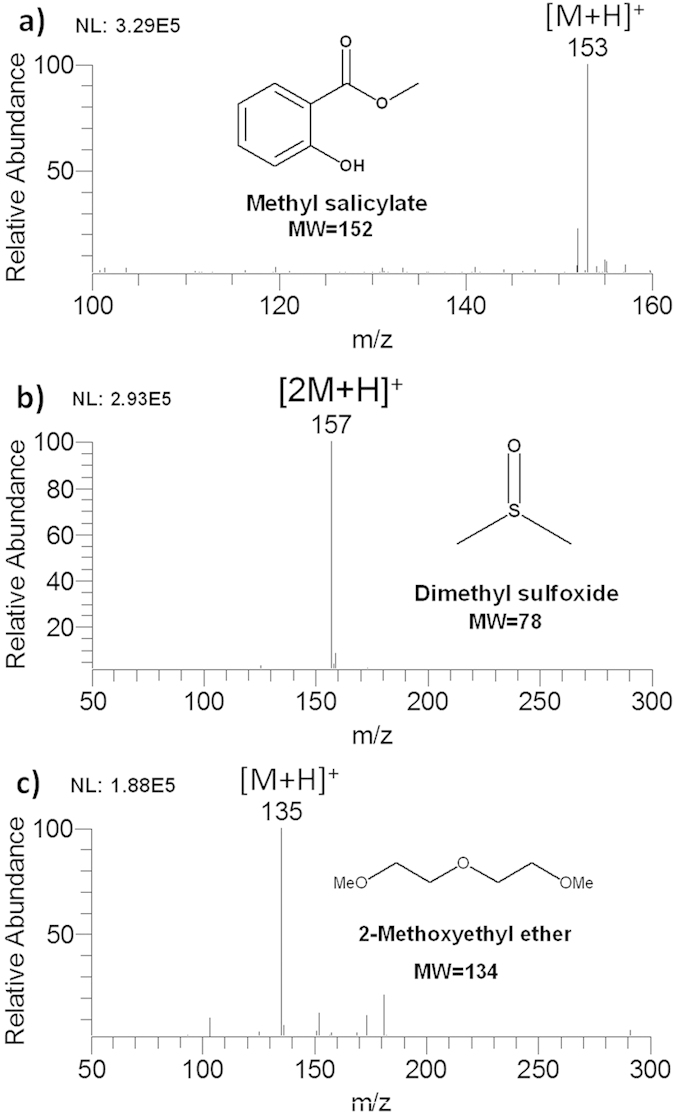
AFI-MS direct analysis of volatile molecules, (**a**) methyl salicylate, (**b**) dimethyl sulfoxide, (**c**) 2-methoxyethyl ether.

Initial experiments were carried out to show the convenience of AFI-MS by direct analysis of trace vapor molecules. The vial containing liquid analyte with low vapor pressure was positioned approximately 1 m away from the AFI and opened for a few seconds to expose trace above vapors to ambient air, while the flame was lit and mass spectrometer kept operating. After several seconds, resulting mass spectra were recorded with high sensitivity. Methyl salicylate, a chemical warfare agent simulant, was detected in its protonated form [M+H]^+^ of *m/z* 153 (Fig. 2a). The signal observed for dimethyl sulfoxide was the protonated dimer (*m/z* 157) as shown in Fig. 2b, whereas for 2-methoxyethyl ether the protonated monomer (*m/z* 135) was mainly observed as shown in Fig. 2c. To demonstrate the stability of AFI-MS in volatile sample analysis, the relative standard deviation (%RSD) and signal/noise (S/N) of dimethyl sulfoxide, 2-methoxyethyl ether and methyl salicylate were provided (see supplementary Table S3). On the other hand, the trace vapor drift introduction mode was compared to the in-flame mode, that is, the sample rod dipped with approximately 0.5 uL dimethyl sulfoxide was subjected directly to flame. Both modes are able to obtain clear mass spectra and ideal signal/noise ratio (supplementary Table S4). These results demonstrate that AFI is able to directly detect trace chemical vapors. Based on this advantage, the AFI has potential to be used for direct and *in-situ* analysis of dangerous chemicals such as chemical warfare agents and toxic industrial chemicals, which pose serious risks to public security and human health. The analytical process of chemical vapors is extremely easy, that is, only a small pot of *n-*butane gas or just an ordinary lighter is required to accomplish ionization without any specialized training. What is more, no signal disturbance and environment pollution occur in the analysis process in terms of solvent-free analysis.

### Liquid sample analysis

**Figure 3:**
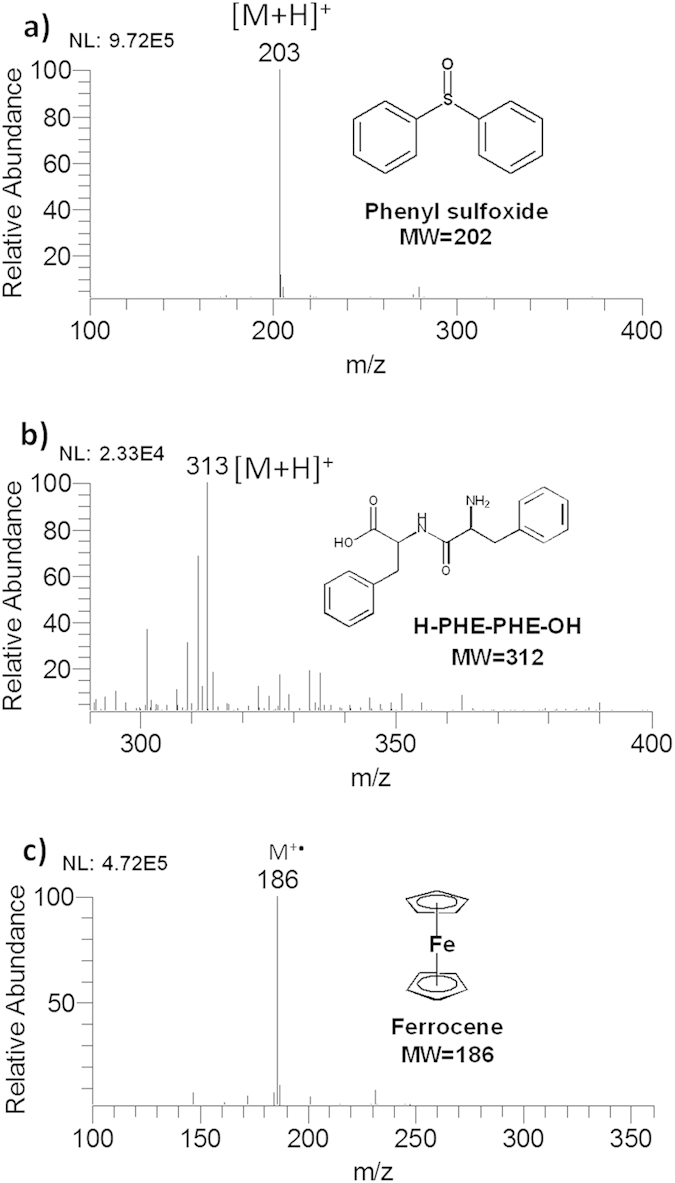
Mass spectra of sample solutions using AFI-MS, (**a**) phenyl sulfoxide, (**b**) H-PHE-PHE-OH, (**c**) ferrocene.

Another paramount application of AFI-MS is direct analysis of diverse solution of organic compounds containing polar, nonpolar, and organometallic compounds. In the analysis of polar and high polar compounds, [M+H]^+^ is normally the primary spectra characteristic, such as 6-chloroguanine, sudan1 and H-ALA-GLY-OH (Supplementary Fig. S4 and Supplementary Fig. S5). Figure 3a showed the signal at *m/z* 203, identified as protoned phenyl sulfoxide with a high-quality mass spectrum. Figure 3b shows the peak of high polar H-PHE-PHE-OH at *m/z* 313, which was detected as a protonated molecule. The AFI can also be able to test organometallic compounds such as ferrocene and nonpolar compounds like anthracene which of the major species shown in the spectra were the molecule ions respectively at *m/z* 186 (Fig. 3c) and *m/z*178 (Supplementary Fig. S6). These findings suggest that AFI is a soft ionization method that M^+^· and [M+H]^+^ are normally the principal spectra characteristic with little or no fragmentation ions. AFI-MS is also capable of direct analysis of solid of organic compounds on the top of sample rod (Supplementary Fig. S7). Because helium and nitrogen might bring the powder samples into mass spectrometer, the direct analysis of solid compounds in powder state using DART is liable to cause contaminations and memory effects. While the analytical process of AFI-MS without assisting gas does not have such problem. What is more, the AFI is simpler and more convenient to perform the *in-situ* and “green” of solvent-free analysis.

### Direct analysis of real-world samples

**Figure 4:**
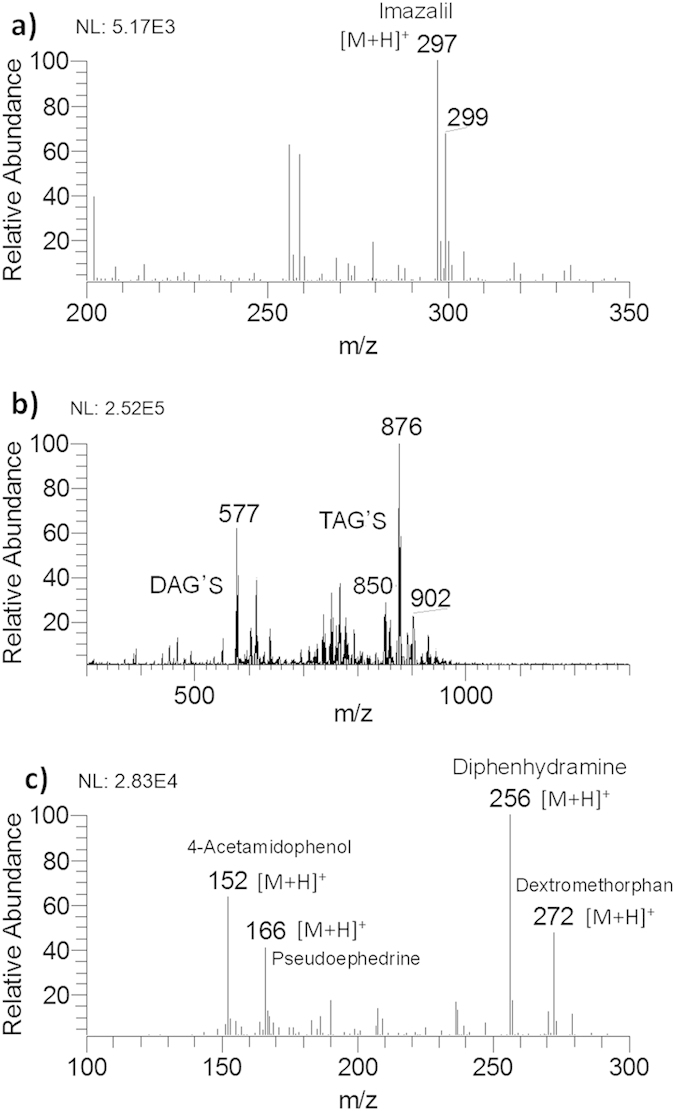
AFI-MS rapid analysis of real-world samples by fast touching samples to the outer flame. (**a**) The peel of apple without any pretreatment. (**b**) Small pieces of pork fat with length 5 mm and width 5mm. (**c**) Drug tablet containing 4-acetamidophenol (325 mg), (1S,2S)-(+)pseudoephedrine hydrochloride (30 mg), *N*-(2-Diphenylmethoxyethyl)-*N*,*N*-dimethylamine hydrochloride (25 mg), dextromethorphan hydrobromide monohydrate (15 mg). Drug tablets were scraped off a thin layer of tablet and expose the subsurface active materials. Four scans were combined to produce the mass spectrum in AFI-MS analysis of this drug tablet.

The capability of AFI was demonstrated by convenient analysis of real-world samples without prior treatment via fast touching the samples in the outer flame. The movie presents a live demonstration of AFI-MS where various real-world samples such as fruit, vegetable, meat, beverage, etc. were rapidly and directly detected while the synchronous computer screen captures the signal responses of mass spectrometry in real time (see Supplementary Movie S1). The food safety issue is crucial for public health and has been focused on momentous attention taking into account ever-increasing food scandals. A vital application of the AFI-MS is direct and rapid analysis of xenobiotics (e.g., illegal additives and pesticide residues) in fruit peels and vegetable surfaces without any treatment and chemical contamination. The susceptible fruit and vegetable are generally treated with fungicides to protect them from rotting for long-time storage^48^,^49^. Figure 4a shows the characteristic isotopic cluster peaks at *m/z* 297 and *m/z* 299, corresponding to protonated imazalil^50^, a kind of widely used fungicide. The confirmation of imazalil was supported by signal accurate mass measurement, the isotopic pattern of two chlorides, and accurate mass element composition (Supplementary Table S7). Animal fat sample was also analyzed directly by AFI. Major constituents of fat are triacylglycerols (TAGs) and their determinations are important due to their vital impact to health^51^,^52^,^53^. Although many technologies are available for determination of TAGs, some of these methods need long and tedious sample pretreatment process^54^,^55^,^56^. Figure 4b showed, as an instance, direct analysis of pork fat using AFI-MS without any pretreatment except cutting fat to pieces. The pivotal finding was that triglycerides were detected (depicted in Supplementary Table S5). Aside from TAGs, some diglycerides (DAGs) were also detected (Supplementary Table S6).

The advantageous applications of AFI-MS in the analysis of real-world samples have been further proved by direct and rapid analysis of active ingredients of pharmaceuticals in the form of tablet, for example, azithromycin dispersible tablets, metronidazole tablets, and compound paracetamol tablets (II) (Supplementary Fig. S8 and Supplementary Fig. S9). In the analysis of tablets containing paracetamol, pseudoephedrine hydrochloride, diphenhydramine hydrochloride and dextromethorphan hydrobromide, all of four active ingredients were simultaneously detected, as shown in Fig. 4c. The peaks at *m/z* 152, 166, 256 and 272 were respectively corresponded to protonated active ingredients of 4-acetamidophenol, pseudoephedrine, diphenhydramine, and dextromethorphan, respectively. The detection of active ingredients in tablets was further confirmed by tandem mass spectrometry (Supplementary Fig. S10), consistent with authentic compounds. In order to explore the analysis sensitivity of AFI-MS, the limit of detection of AFI-MS in detection of propyphenazone was obtained at 1 picogram level (see Supplementary Fig. S11). AFI can be also used to direct detect allicin, a primary thiosulfate in the garlic. The peaks at *m/z* 163 and m/z 180, respectively corresponded to [M+H]^+^ and [M+NH_4_]^+^ (see Supplementary Fig. S13). These results shown here demonstrate that analytical procedures of real-world samples are solvent-free, that is, without any chemical contamination, which presents a “green” analytical process.

### Analysis of the sample in the negative ion mode

After testing the application of AFI-MS in positive ion mode, the negative ion mode was also performed. The performance of AFI-MS in negative ion mode is not good as the positive ion mode. For instance, only weak signal of [M-H]^–^ at *m/z* 139 (Supplementary Fig. S14) was obtained from AFI-MS analysis of 3-fluorobenzoic acid. It is possible that the negative ions with lone pair electrons are not stable in oxidation environment of AFI-MS. We are still working to search a better condition for AFI-MS analysis of organic compound in negative ion mode.

## Discussion

A novel ambient ionization technique, ambient flame ionization source, has been developed for only requirement of ambient flame without other assistances. The ionization process in the AFI is gentle, generating charged intact molecular species with rare or no fragmentation ions. In addition, AFI is compatible with other atmospheric pressure ionization mass spectrometer, such as triple-quadrupole mass spectrometer (Supplementary Fig. S15). The unique characters of AFI-MS relied on the following critical points: the small flame by the combustion of *n-*butane in open air condition provides a focused heating zone (about 500 ^o^C) for fast desorption of organic compounds and meanwhile the gaseous reactive species from the flame rapidly promoted the spontaneous ionization of the organic compound. Of course there are some potential competing processes might occur at same time, such as thermal degradation, oxidation, pyrolysis of analytes. Therefore, in order to get the better results and controlling side effects of competing oxidation and degradation, many parameters of AFI-MS could be optimized, such as: the choice of fuel, fuel flow, size of flame, sample introduction mode, sample introduction point in the flame and the distance between the flame and the inlet of MS.

AFI-MS technology have some similarity and differences with some previously reported thermal desorption/ionization techniques such as, ambient pressure thermal desorption ionization (APTDI)^25^, and pyrolysis mass spectrometry (PyMS)^57^. The APTDI is a popular ambient ionization method by rapid heating of the sample with the help of heated nitrogen gas flow (100 ~ 450 ^o^C) for transferring the generated ions to the mass spectrometer^25^,^26^. The pyrolysis mass spectrometry (PyMS) mainly studies the decomposition products of macromolecular samples in high temperature pyrolysis (even to 600 ^o^C) condition by suitable mass spectrometry methods. The similar point is that high temperature of outer flame (about 500 ^o^C when *n*-butane was applied as fuel) in AFI-MS was also important factor to achieve rapid desorption of the organic compounds, but the different points are that the reactive species in the *n-*butane flame might be critical for the ionization process of AFI-MS. In the process of AFI-MS analysis, the melting and boiling phenomena were not obvious, but it is believed that they are important factors for sample desorption step. The reasons might be that: (1) the high sensitivity of AFI-MS required only small or trace amount of samples, (2) the focused heating zone of outer flame (about 500 ^o^C) could provide enough energy in very short time to initiate the fast desorption and ionization of analytes. Most organic compounds responded fast in AFI-MS analysis, and we found that normally the volatile organic compounds responded slightly faster than the solid samples with higher melt and boil point. In the AFI-MS analysis, the fast touching of samples with outer flame zone usually was applied in very short time usually at about 1–3 seconds.

The experimental results showed that the AFI-MS was an ideal method for mass spectrometric analyses of organic compounds and the applicability of AFI-MS technique has close relationship with the physicochemical properties of the analytes. Because the fast sample desorption process in the outer flame has close relationship to the melting and boiling point and the vapour pressure in ambient condition of the analyte. The ionization process of the gaseous analyte mostly depended on the ionization energy and proton affinity. The capabilities of AFI are demonstrated through rapid, direct, *in-situ* mass spectrometric analysis of sample in various states, such as volatile organic compounds (e.g. phenyl sulfoxide, 6-chloroguanine), medicine molecules in tables (e.g. azithromycin dispersible tablets, metronidazole tablets), amino acids, lipid molecules (e.g. pork fat). Because most of these organic compounds could be desorbed and ionized with the help of flame, most of these organic compounds were protonated in AFI-MS; however, the compound with low ionization energy, such as ferrocene (6.82 eV)^58^, anthracene (7.439 eV)^59^ give radical cations. All data demonstrated that AFI-MS is a promising approach in the related research area since it is fast, simple, and also carries great potential for portability. As such, AFI-MS will be of interest to the analytical community at large, such as public security, environmental protection, therapeutic drug monitoring, and food quality monitoring. Researches on the complicated combustion process in AFI-MS are still going on to harness its advantages for optimizing AFI-MS conditions and to reduce the competing side-processes, such as: degradation, oxidation, pyrolysis and combustion of analytes. In view of convenience and versatility, the AFI is potentially an attractive candidate to couple portable or miniature mass spectrometry to perform in-field analysis of target samples in their undisturbed environment and native states. Such simple and high-efficiency AFI method might have a bright future in direct, high-throughput, and *on-site* analysis.

## Methods

### Sample collection

Chemical reagents were directly used without any further purification. Food was purchased from local stores without any further treatment. All of food was directly exposed to the flame without any treatment. Drug tablets were bought from local pharmacy. Coated tablets needed to scrape off a thin layer of the tablet and expose the subsurface active materials, whereas uncoated tablets were directly detected without any treatment. (See the Supplementary Information)

### AFI-MS

Experiments were performed on a liner ion trap Fourier transform-ion cyclotron resonance ULTRA XL mass spectrometer (Thermo Fisher Scientific) and Finnigan TSQ Quantum Access™ triple-quadrupole mass spectrometer (Thermo Fisher Scientific, Waltham, MA) installed with a homemade AFI ion source. The basic operation conditions of a liner ion trap fourier transform-ion cyclotron resonance ULTRA XL mass spectrometer were set as follow: capillary voltage: 9 V; the capillary temperature, 250 ^o^C; tube lens voltage: 100 V. The ion optics conditions were set as follow: multipole 00 offset voltage, −4 V; multipole 0 offset voltage, −4.5 V; multipole 1 offset voltage, −15.5 V; lens 0 voltage, −4.5 V; lens 1 voltage, −40.0 V; gate lens voltage, −48.0 V; front lens voltage, −5.5 V. Peak integration and data acquisition were performed through the instrument embedded Xcalibur® software. The optimal distance between the center of butane flame and the inlet to mass spectrometer is 1 cm (Supplementary Fig. 2a). The AFI-MS experiments could also performed in Thermo TSQ Quantum AccessTM triple-quadrupole mass spectrometer (Thermo-Fisher Scientific, Waltham, USA) (Supplementary Figure S15).

## Additional Information

**How to cite this article**: Liu, X.-P. *et al.* Direct and Convenient Mass Spectrometry Sampling with Ambient Flame Ionization. *Sci. Rep.* **5**, 16893; doi: 10.1038/srep16893 (2015).

## Supplementary Material

Supplementary Information

Supplementary Information
