# Supplementary material for: Direct and Convenient Mass Spectrometry Sampling with Ambient Flame Ionization
Source: Sci Rep. 2015 Nov 19;5:16893. doi: 10.1038/srep16893 (PMC4652273; doi:10.1038/srep16893)
Supplement: Supplementary Information [file srep16893-s1.doc]

Supplementary Information

Direct and Convenient Mass Spectrometry Sampling with Ambient Flame Ionization

Xiao-Pan Liu, Hao-Yang Wang*, Jun-Ting Zhang, Meng-Xi Wu, Wan-Shu Qi, Hui Zhu and Yin-Long Guo*

National Center for Organic Mass Spectrometry in Shanghai,

State Key Laboratory of Organometallic Chemistry,

Shanghai Institute of Organic Chemistry

Chinese Academy of Sciences

345 Lingling Road, Shanghai 200032, P. R. China.

*E-mail: haoyangwang@sioc.ac.cn (H.-Y. Wang).

*E-mail: ylguo@sioc.ac.cn (Y.-L. Guo).

**Table of Contents**

**1. Materials and chemicals.....................................................................................................................S3**

**2. AFI-MS experimental conditions.................................................................................................S4-S7**

**3. AFI-MS analysis of vapor molecules..........................................................................................S7**

**4. AFI-MS analysis of solutions of organic compounds.................................................................S8-S9**

**5. AFI-MS analysis of solid of organic compounds............................................................................S10**

**6.** **AFI-MS analysis of real-world samples..................................................................................S11-S16**

**1) AFI-MS analysis of active ingredients of pharmaceuticals...............................................S11-S14**

**2) AFI-MS analysis of pork fat.................................................................................................S15-p16**

**3) AFI-MS analysis of garlic............................................................................................................S16**

**7.** **AFI-MS analysis in the negative ion mode.....................................................................................S17**

1. **AFI analysis by triple quadrupole mass spectrometer................................................................S18**
2. **Table.................................................................................................................................................S19**
3. **Materials and chemicals**

Chemical reagents were directly used without any further purification. Methyl salicylate, dimethyl sulfoxideand, phenyl sulfoxide, 2-methoxyethyl ether, 5,6-dimethylbenzimidazole, 6-chloroguanine, sudan1, were bought from J&K scientific LTD (China). H-PHE-PHE-OH, H-ALA-GLY-OH, ferrocene and 3-fluorobenzoic acid were purchased from Sigma-Aldrich (Germany). HPLC-grade acetonitrile and water were provided by Merck (Darmstadt, Germany) and used to prepare sample solution (V_H2O_:V_CH3CN_=1:1).

Food was purchased from local stores without any further treatment. Drug tablets were bought from local pharmacy. Coated tablets needed to scrape off a thin layer of the tablet and expose the subsurface active materials, whereas uncoated tablets were directly detected without any treatment.

1. **AFI-MS experimental conditions**

Experiments were performed on a liner ion trap fourier transform-ion cyclotron resonance ULTRA XL mass spectrometer (Thermo Fisher Scientific). The basic operation conditions were set as follow: capillary voltage: 9V; the capillary temperature, 250^o^C; tube lens voltage: 100V. The ion optics conditions were set as follow: multipole 00 offset voltage, -4V; multipole 0 offset voltage, -4.5V; multipole 1 offset voltage, −15.5V; lens 0 voltage, −4.5 V; lens 1 voltage, −40.0 V; gate lens voltage, −48.0 V; front lens voltage, −5.5 V. Peak integration and data acquisition were performed through the instrument embedded Xcalibur® software. The time scale of each scan is normally 500ms in the experimental condition and the scan time can also be varied according the specific requirements. The AFI-MS experiments could also performed in Thermo TSQ Quantum Access^TM^ triple-quadrupole mass spectrometer (Thermo-Fisher Scientific, Waltham, USA). Data acquisition and analysis were carried out with the Xcalibur software package (Version 2.0, Thermo Fisher Scientific). The basic operation conditions were vacuum, 2.3×10^–6^ Torr; the capillary temperature, 275^o^C. Supplementary Figure S15 showed the AFI mass spectra obtained with triple-quadrupole mass spectrometer.

The flow rate of *n-*butane was measured by soap-film flow meter (Shenli company, China). The flame temperature of different points in flame (Supplementary Table S2) were acquired by contact thermocouple (Jingdayi company, China). The optimal distance between the center of flame and the inlet to mass spectrometer is 1cm and the length of flame is approximately 1cm. In the process of analysis, samples were directly subjected to the outer flame, which was liable to sufficient contact with active species in the flame. All of food was directly exposed to the outer flame without any treatment.Flames of different fuels were compared, such as ethyl acetate, ethanol and acetone (Supplementary Figure S2). The experimental results demonstrate that the optimal fuel was *n*-butane. The lighter is also able to perform ambient flame ionization (see Supplementary Fig.3), the lighter is easy to carry and operate, lightweight, universally available and convenient, especially combining with portable mass spectrometry for in-situ and in-field analysis.


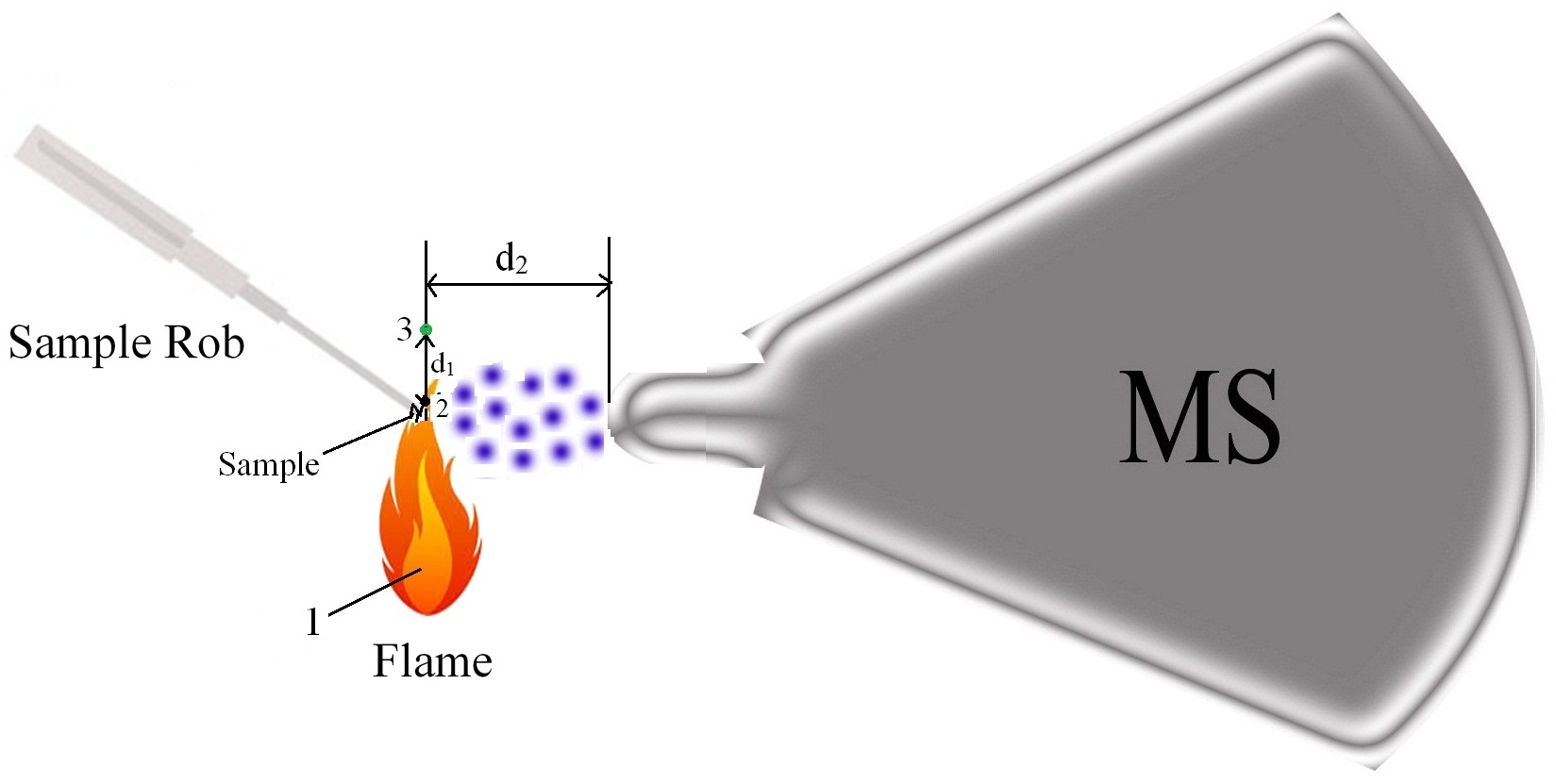


**Supplementary Fig. S1:** The schematic diagram of AFI-MS. 1. Inner flame; 2. the sample introduction position outer flame; 3. top of the flame. d_1_=3mm (the distance between position 2 and position 3), d_2_=10mm (the distance between position 2 and the inlet of mass spectrometer).The relationship of flame sizes and temperatures in different positions of flame (position 1, 2 and 3) with the *n*-butane flow rate were summarized in Supplementary Table S2. In most of our experiments, the sample rod with sample doped on the glass top was put in the position 2 for fast touching analysis. The diameter of outlet of the *n*-butane transferring pipe is 0.9mm.


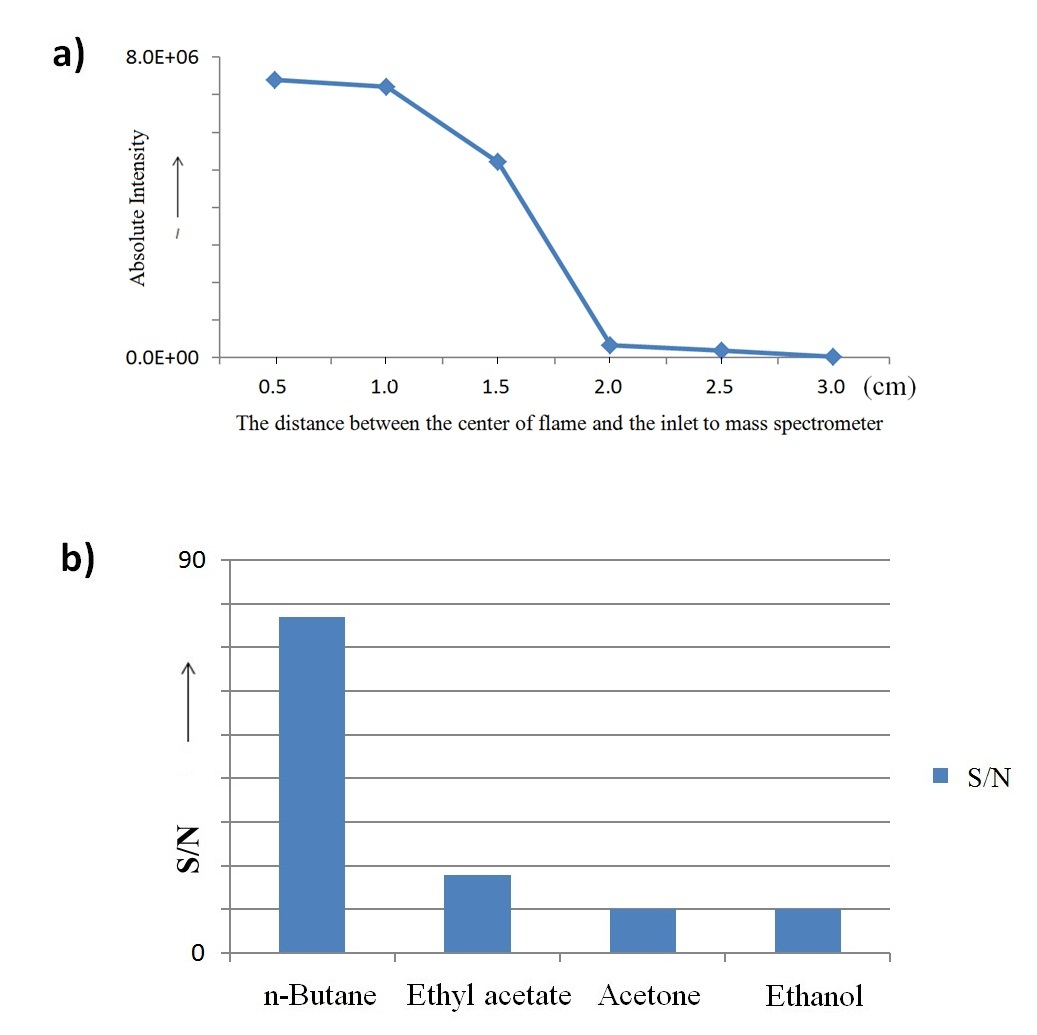


**Supplementary Figure S2**: a) Effects of the distance between the position 2 of outer flame (Supplementary Fig. S1) and the inlet to mass spectrometer on the signal intensity of phenyl sulfoxide. b) The signal/noise(S/N) of phenyl sulfoxide in the outer flame of different fuels. Phenyl sulfoxide was dissolved in the mixed solvents (V_H2O_:V_CH3CN_=1:1). The concentration of phenyl sulfoxide was 0.1 mg mL^-1^. The sample rob dipped approximately 0.5uL solution of compound directly was subjected to flame.

**Supplementary Table S1**. The intensity repeatability (%RSD) and signal/noise(S/N) for the signals of phenyl sulfoxide in different introduction points using AFI-MS analysis (the number of replicates is three).

| Introduction points | Intensity | Signal/Noise | Intensity repeatabilty (%RSD, n=3) |
| --- | --- | --- | --- |
| Top of the flame (position 3 in Fig. S1) | 1.46×10^5^ | 63 | 11.9 |
|  | 1.80×10^5^ | 75 |  |
|  | 1.82×10^5^ | 82 |  |
| Outer flame  (position 2 in Fig. S1) | 1.68×10^6^ | 86 | 8.08 |
|  | 1.43×10^6^ | 71 |  |
|  | 1.54×10^6^ | 78 |  |
| Inner flame  (position 1 in Fig. S1) | 1.25×10^5^ | 26 | 33.4 |
|  | 2.53×10^5^ | 33 |  |
|  | 2.21×10^5^ | 42 |  |

**Supplementary Table S2**. The relationship of the flame sizes and temperatures of the different positions of flame with flow rate of *n-*butane(the number of replicates is three) .

| Size of the flame | Outer flame  (*^o^C*) | Inner flame  (*^o^C*) | top of flame end (*^o^C*) | flow rate  (mL/min) |
| --- | --- | --- | --- | --- |
| 1.0 cm | 468 ±10.3 | 321 ±13.3 | 363±15.3 | 15.1 |
| 1.5 cm | 493±12.2 | 355±15.0 | 390±14.5 | 18.5 |
| 2.0 cm | 571±17.5 | 386±15.1 | 441±15.5 | 23.8 |


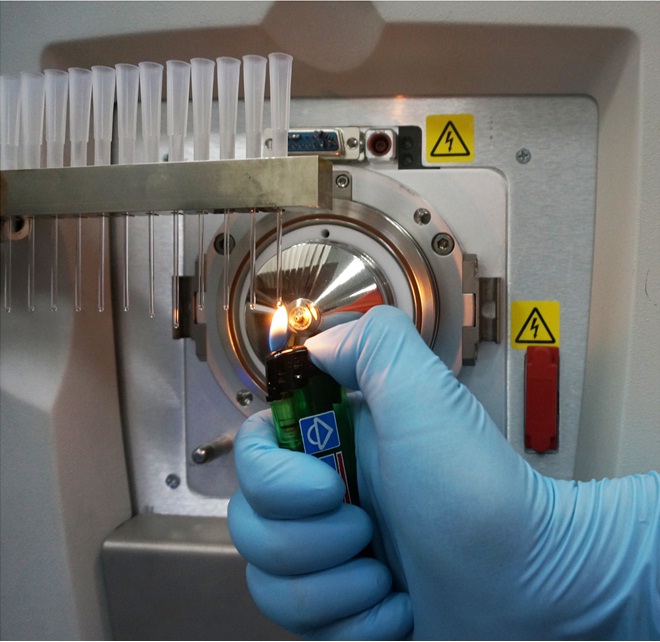


**Supplementary Figure S3:** Photograph of lighter as an ambient ionization source for analysis of samples. The sample rob dipped approximately 0.5uL solution of compound was directly subjected to the flame of lighter.

1. **AFI-MS analysis of vapor molecules**

**Supplementary Table S3**. The intensity repeatability (%RSD) and signal/noise(S/N) of vapour samples in AFI-MS analysis (the number of replicates is three).

| Compounds | [M+H]^+^ | Intensity | Signal/Noise | Intensity repeatability  (%RSD, n=3) |
| --- | --- | --- | --- | --- |
| 2-Methoxyethyl ether | *m/z* 135 | 1.79×10^4^ | 54 | 11.7 |
|  |  | 1.68×10^4^ | 43 |  |
|  |  | 1.42×10^4^ | 32 |  |
| Dimethyl sulfoxide | *m/z* 157 | 9.34×10^4^ | 68 | 5.90 |
|  |  | 1.01×10^5^ | 84 |  |
|  |  | 1.05×10^5^ | 123 |  |
| Methyl salicylate | *m/z* 153 | 4.27×10^3^ | 29 | 10.9 |
|  |  | 4.62×10^3^ | 38 |  |
|  |  | 3.71×10^3^ | 48 |  |

**Supplementary Table S4**. The intensity repeatability (%RSD) and signal/noise(S/N) of dimethyl sulfoxide vapor in drift introduction mode and in-flame mode using AFI-MS analysis (the number of replicates is three).

| Mode of analyses | Intensity | Signal/Noise | Intensity repeatability  (%RSD, n=3) |
| --- | --- | --- | --- |
| Trace vapor drift introduction mode | 9.34×10^4^ | 68 | 5.90 |
|  | 1.01×10^5^ | 84 |  |
|  | 1.05×10^5^ | 123 |  |
| In-flame mode | 1.44×10^5^ | 91 | 6.82 |
|  | 1.57×10^5^ | 110 |  |
|  | 1.65×10^5^ | 175 |  |

1. **AFI-MS analysis of solutions of organic compounds.**

AFI-MS is able to direct analysis of diverse organic compounds containing polar, nonpolar, and organometallic compounds. HPLC-grade acetonitrile and water were provided by Merck (Darmstadt, Germany) and used to prepare sample solution (V_H2O_:V_CH3CN_=1:1). Concentrations of all of organic compounds were 0.1mg mL^-1^. The sample rob dipped approximately 0.5uL solution of compound was directly subjected to the outer flame.


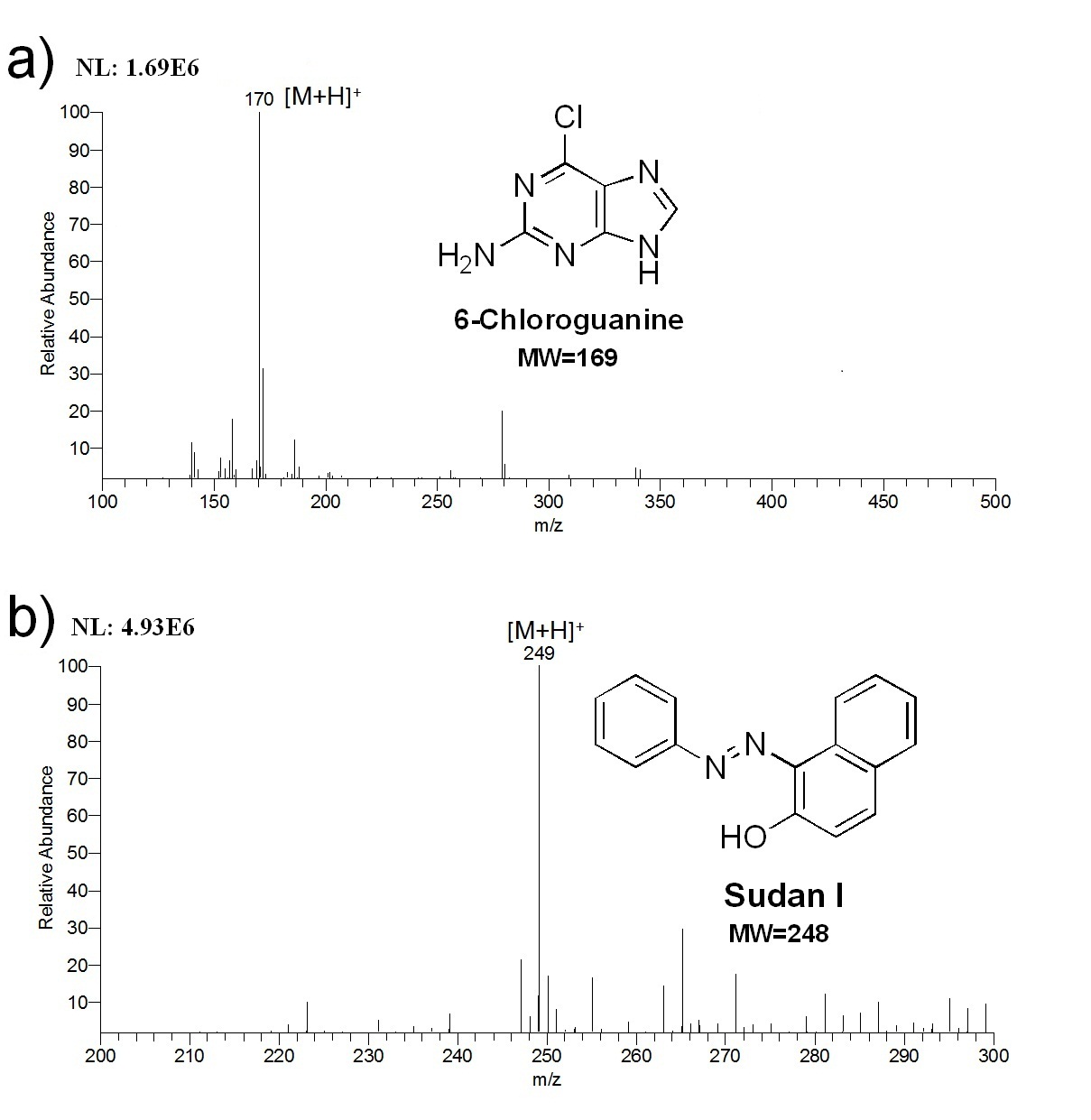


**Supplementary Figure S4**: AFI-MS spectra in analysis of polar organic compounds: a) 6-Chloroguanine. b) Sudan 1. The compounds were dissolved in the mixed solvents (V_H2O_:V_CH3CN_=1:1). The concentrations of compounds were 0.1 mg mL^-1^. The sample rob dipped approximately 0.5uL solution of compound directly was subjected to outer flame.


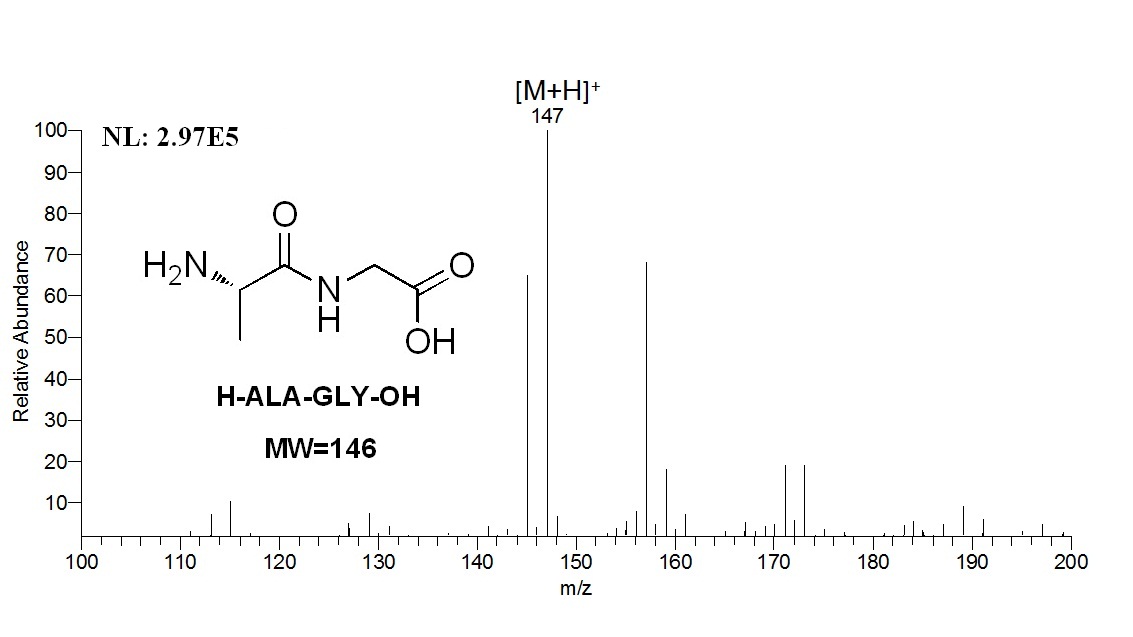


**Supplementary Figure S5**: AFI-MS spectrum in analysis of high-polar H-ALA-GLY-OH. H-ALA-GLY-OH was dissolved in the mixed solvents (V_H2O_:V_CH3CN_=1:1). The concentration was 0.1 mg mL^-1^. The sample rob dipped approximately 0.5uL solution of H-ALA-GLY-OH was directly subjected to outer flame.


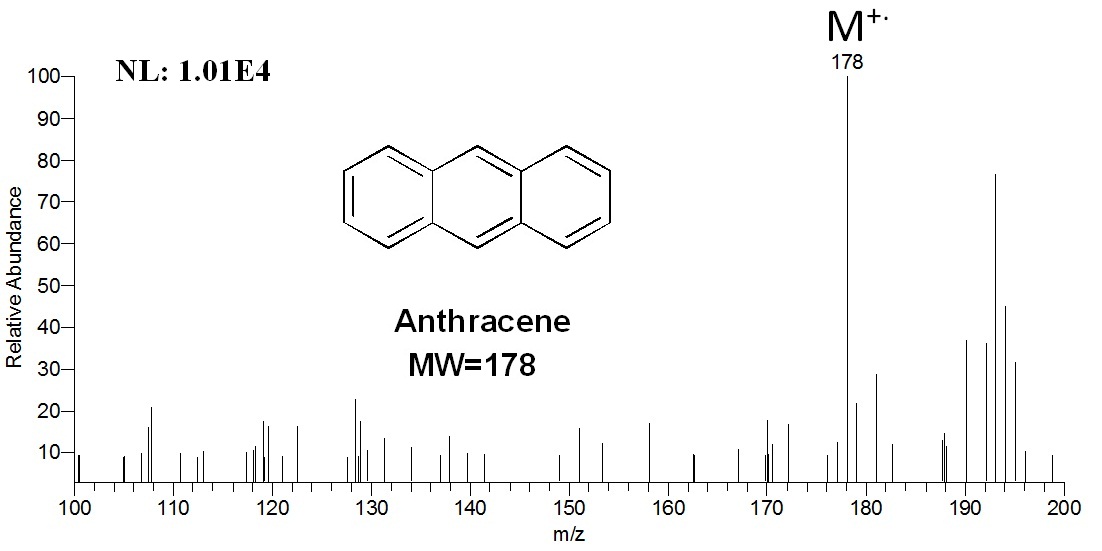


**Supplementary Figure S6**：AFI-MS spectrum in analysis of anthracene. The anthracene was dissolved in the dichloromethane. The concentration of anthrance was 0.1 mg mL^-1^. The sample rob dipped approximately 0.5uL solution of anthracene was directly subjected to the outer flame.

1. **AFI-MS analysis of solid of organic compounds.**


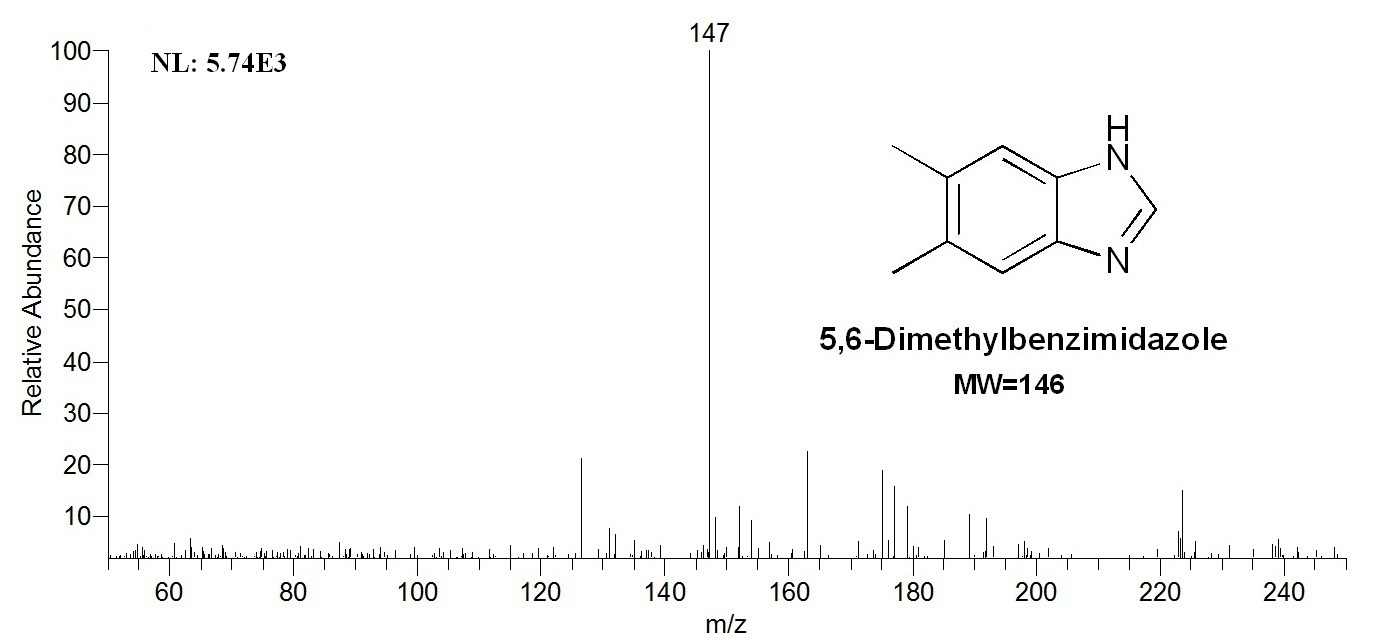


**Supplementary Figure S7**：AFI-MS spectrum in direct analysis of solid sample 5,6-dimethylbenzimidazole (approximately 500ng) on the sample rod.

1. **AFI-MS direct analysis of real-world samples**

AFI-MS has successfully applied to analyze the organic compounds in diverse real-world samples such as active ingredients of pharmaceuticals, fruit, vegetables, meat and garlic without sample pretreatments.

1. **AFI-MS direct analysis of active ingredients of pharmaceuticals**

The advantageous application of AFI-MS in the analysis of real-world samples has been further proved by direct and rapid detection of active ingredients of pharmaceuticals in the form of tablet. Coated tablets needed to scrape off a thin layer of the tablet and expose the subsurface active materials, whereas uncoated tablets were directly detected.


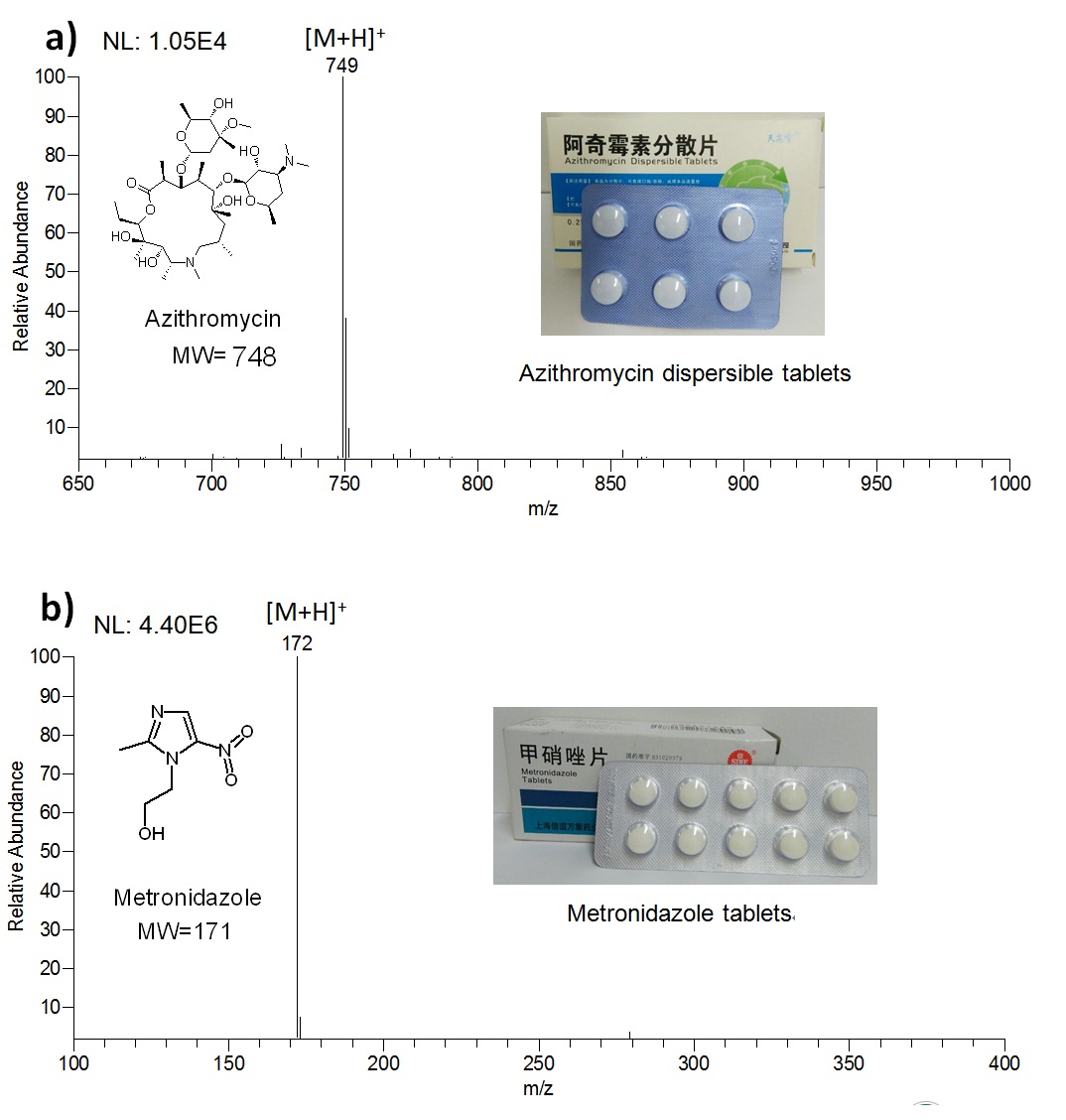


**Supplementary Figure S8**: AFI-MS spectra in analysis of a) Azithromycin dispersible tablets (250mg), and b) Metronidazole tablets (200mg). The tablets were directly exposed to outer flame.


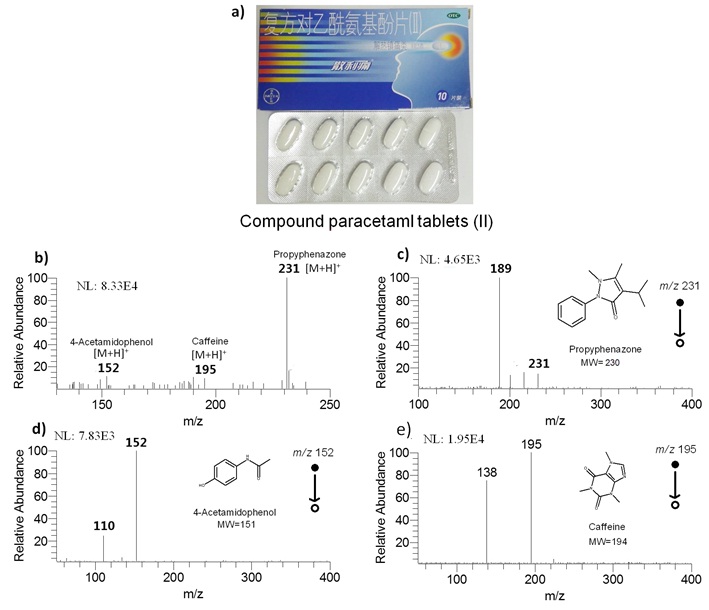


**Supplementary Figure S9**: AFI-MS analysis of compound paracetamol tablets (II) containing 4-acetamidophenol (250 mg), propyphenazone (150 mg), caffeine (50 mg). a) The photograph of compound paracetamol tablets (II), b) AFI-MS spectrum of active ingredients in compound paracetamol tablets (II), AFI-MS/MS spectra of c) protonated propyphenazone at *m/z* 231, d) protonated 4-acetamidophenol at *m/z* 152, e) protonated caffeine at *m/z* 195. The tablets were directly exposed to the flame.


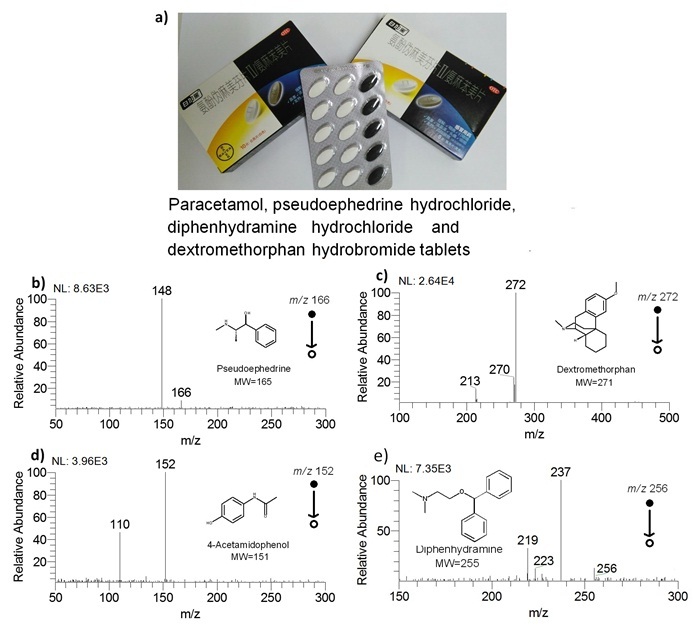


**Supplementary Figure S10**: AFI-MS analysis of paracetamol, pseudoephedrine hydrochloride, diphenhydramine hydrochloride and dextromethorphan hydrobromide tablets. a) The photograph of tablets, AFI-MS/MS spectra of b) protonated pseudoephedrine at *m/z* 166, c) protonated dextromethorphan at *m/z* 272, d) protonated 4-Acetamidophenol at *m/z* 152, and e) protonated diphenhydramine at *m/z* 256.

In the AFI-MS analysis of propyphenazone, three times were performed to obtain LOD. The amount of propyphenazone was 1 picogram and the related mass spectra and the S/N values were listed below.


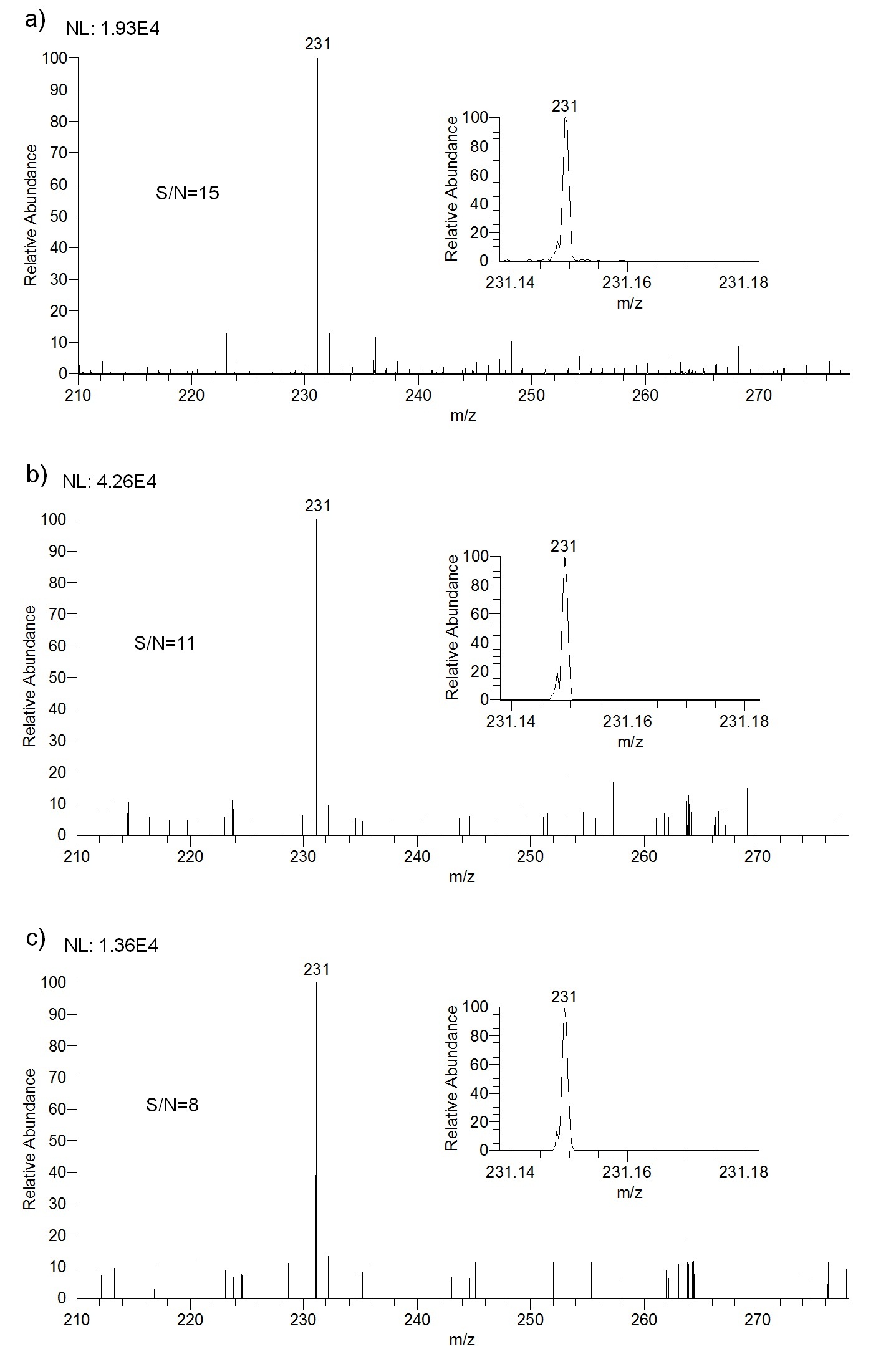


**Supplementary Figure S11:** AFI-MS spectra in analysis of propyphenazone. Acetonitrile and water were used to prepare sample solution (V_H2O_:V_CH3CN_=1:1). The concentration of propyphenazone was 1ng/mL. The sample rob dipped with 1uL solution of propyphenazone was directly subjected to flame. The inserted figure was the expanded signal at *m/z* 231.

**2) AFI-MS direct analysis of pork fat**

1) AFI can directly analyze pork fat, primary detected ions are TAGs and DAGs. Because compounds of fat are extremely complex, partly TAGs and DAGs are listed in Tablet S1 and Tablet S2. In some cases not the only one TAG and DAG could be ascribed to an elemental composition estimated based on the accurate mass of measured signals given that different fatty acids link to the glycerol backbone.


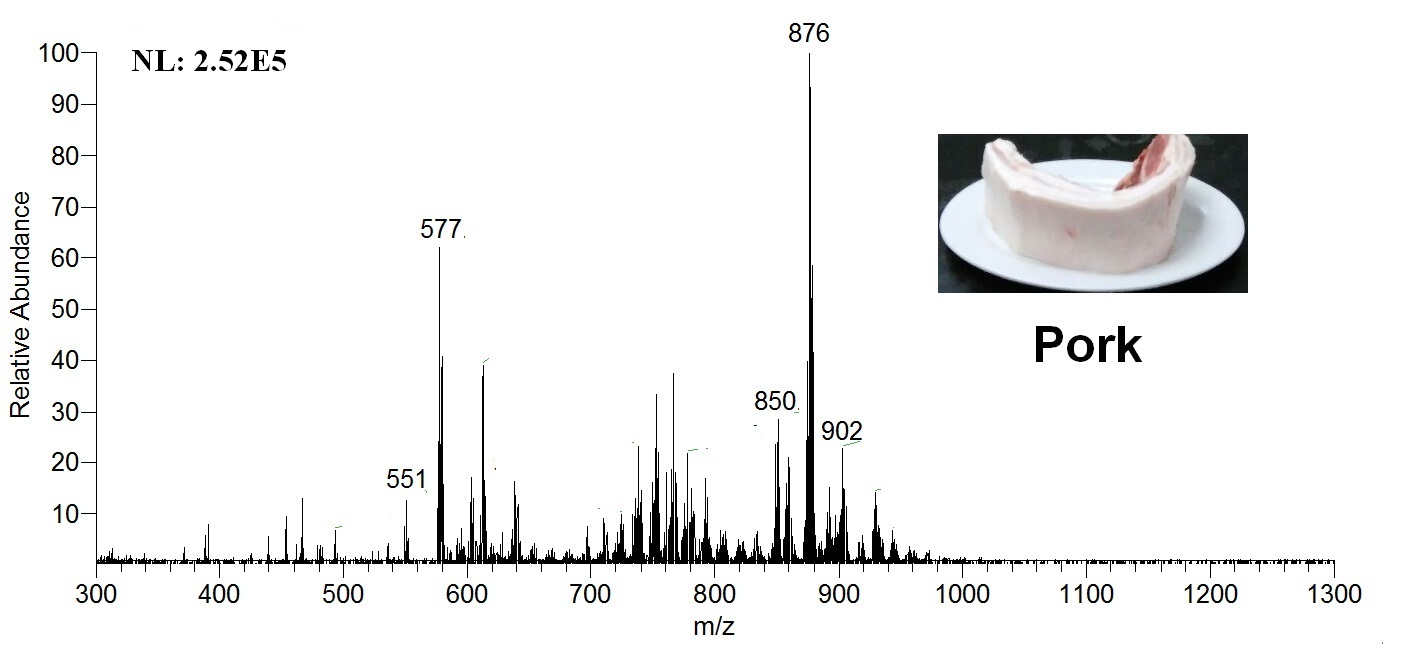


**Supplementary Figure S12**: AFI-MS analysis of the pork fat without any pretreatment except cutting fat to pieces.

**Supplementary Table S5**. Principal TAGs identified in the pork fat using AFI-MS

| TAG^a^ | Elemental compositions | CN/DB^b^ | [M+H]^+^ | | Relative error(ppm) | [M+NH_4_]^+^ | | Relative error(ppm) |
| --- | --- | --- | --- | --- | --- | --- | --- | --- |
|  |  |  | Detected *m/z* | Calculated *m/z* |  | Detected *m/z* | Calculated *m/z* |  |
| GOLa/OOM/POPo/PLP/SLM | C_53_H_98_O_6_ | 53:5 | 831.7431 | 831.7436 | -0.6 | 848.7697 | 848.7702 | -0.6 |
| AOLa/POP/SOM | C_53_H_100_O_6_ | 53:4 | 833.7602 | 833.7593 | 1.1 | 850.7858 | 850.7858 | 0.0 |
| OOPo/OLP/SLnP | C_55_H_100_O_6_ | 55:6 | 857.7595 | 857.7593 | 0.2 | 874.7844 | 874.7858 | -1.6 |
| GOM/OOP/SLP | C_55_H_102_O_6_ | 55:5 | 859.7750 | 859.7749 | 0.1 | 876.7997 | 876.8015 | -2.1 |
| SOP/AOM/BOLa/BLLa | C_55_H_104_O_6_ | 55:4 | 861.7929 | 861.7906 | 2.7 | 878.8163 | 878.8171 | -0.9 |
| OOO/SLnS | C_57_H_104_O_6_ | 57:6 | 885.7847 | 885.7906 | -6.7 | 902.8158 | 902.8171 | -1.4 |

**Supplementary Table S6**. Principal DAGs identified in the pork fat using AFI-MS

| DAG^a^ | Elemental compositions | CN/DB^b^ | Detected *m/z*  [M-H_2_O+H]^+^ | Calculated *m/z* | Relative error(ppm) |
| --- | --- | --- | --- | --- | --- |
| PPo/MO | C_35_H_66_O_5_ | 35:3 | 549.4879 | 549.4877 | 0.4 |
| MS/PP | C_35_H_68_O_5_ | 35:2 | 551.5037 | 551.5034 | 0.5 |
| PO/PoS | C_37_H_70_O_5_ | 37:3 | 577.5189 | 577.5190 | -0.2 |
| PS | C_37_H_72_O_5_ | 37:2 | 579.5346 | 579.5347 | -0.2 |
| LO | C_39_H_70_O_5_ | 39:5 | 601.5185 | 601.5190 | -0.8 |
| LS/OO | C_39_H_72_O_5_ | 39:4 | 603.5347 | 603.5347 | 0.0 |
| SO | C_39_H_74_O_5_ | 39:3 | 605.5504 | 605.5503 | 0.2 |

^a^The abbreviations of fatty acids in TAGs and DAGs of studied pork fat: L, linoleic acid (C18:2); O, oleic acid (C18:1); P, pamitic acid (C16:0); Ma, margaric acid (C17:0); Po, palmitoleic acid (C16:1); Ln, linolenic acid (C18:3)；A, arachidic acid (C20:0); S, stearic acid (C18:0); La, lauric acid (C12:0). M, myristic acid (C14:0); G, gadoleic acid (C20:1); B, behenic acid (C22:0); Ma, margaric acid (C17:1). ^b^carbon number (CN) : double bond number (DB).

1. **AFI-MS direct analysis of garlic**


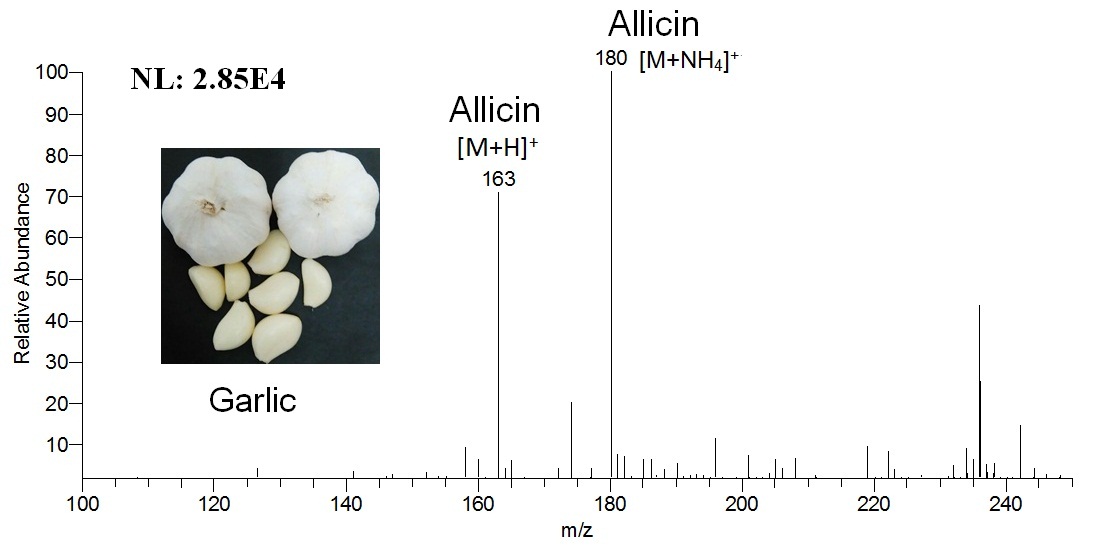


**Supplementary Figure S13**: AFI-MS analysis of garlic clove without any treatment except scripping the surface peel. Allicin, a primary thiosulfate in the garlic, was detected at *m/z* 163 and m/z 180, corresponding to [M+H]^+^ and [M+NH_4_]^+^

1. **AFI-MS analysis in the negative ion mode**


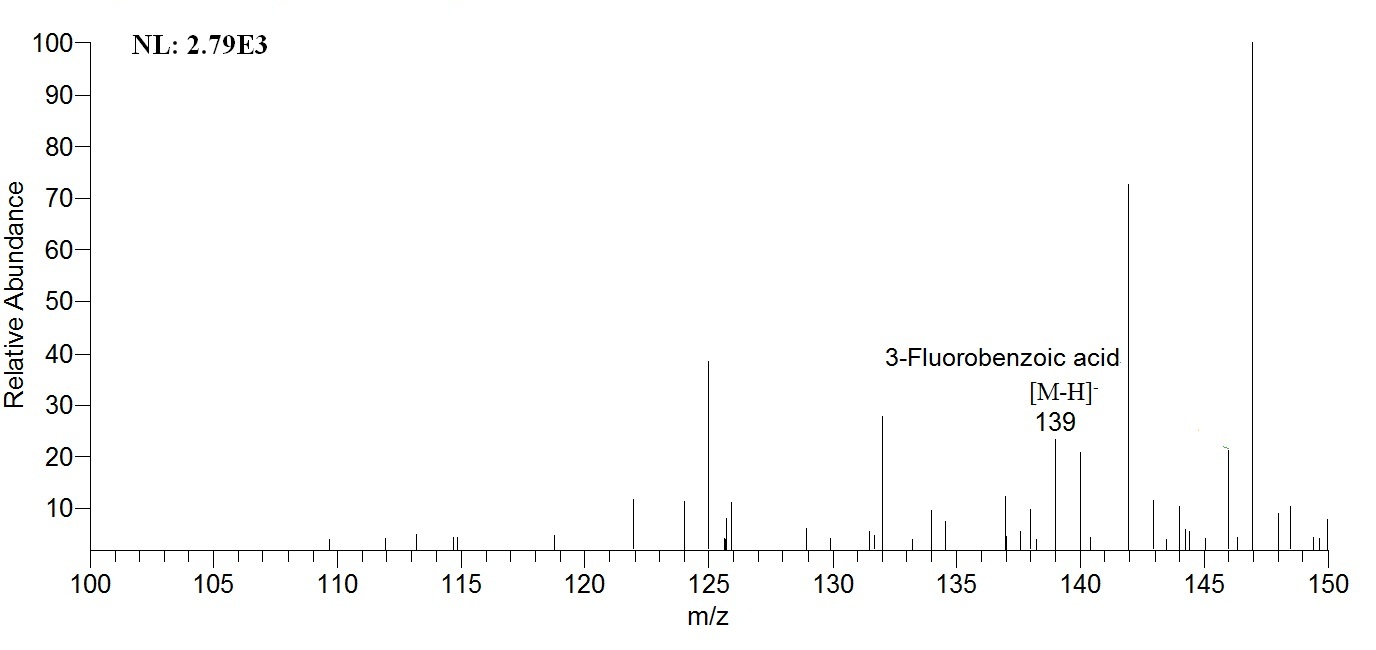


**Supplementary Figure S14**：AFI-MS analysis of 3-fluorobenzoic acid in the negative mode. The 3-fluorobenzoic acid was dissolved in the mixed solvents (V_H2O_:V_CH3CN_=1:1). The concentration of anthrance was 0.1 mg mL^-1^. The sample rob dipped approximately 0.5uL solution of anthracene was directly subjected to the flame.

1. **AFI analysis by triple-quadrupole mass spectrometer**

AFI is compatible with almost all of atmospheric pressure ionization mass spectrometers, for example triple-quadrupole mass spectrometer. High-quality mass spectra can be obtained.

**
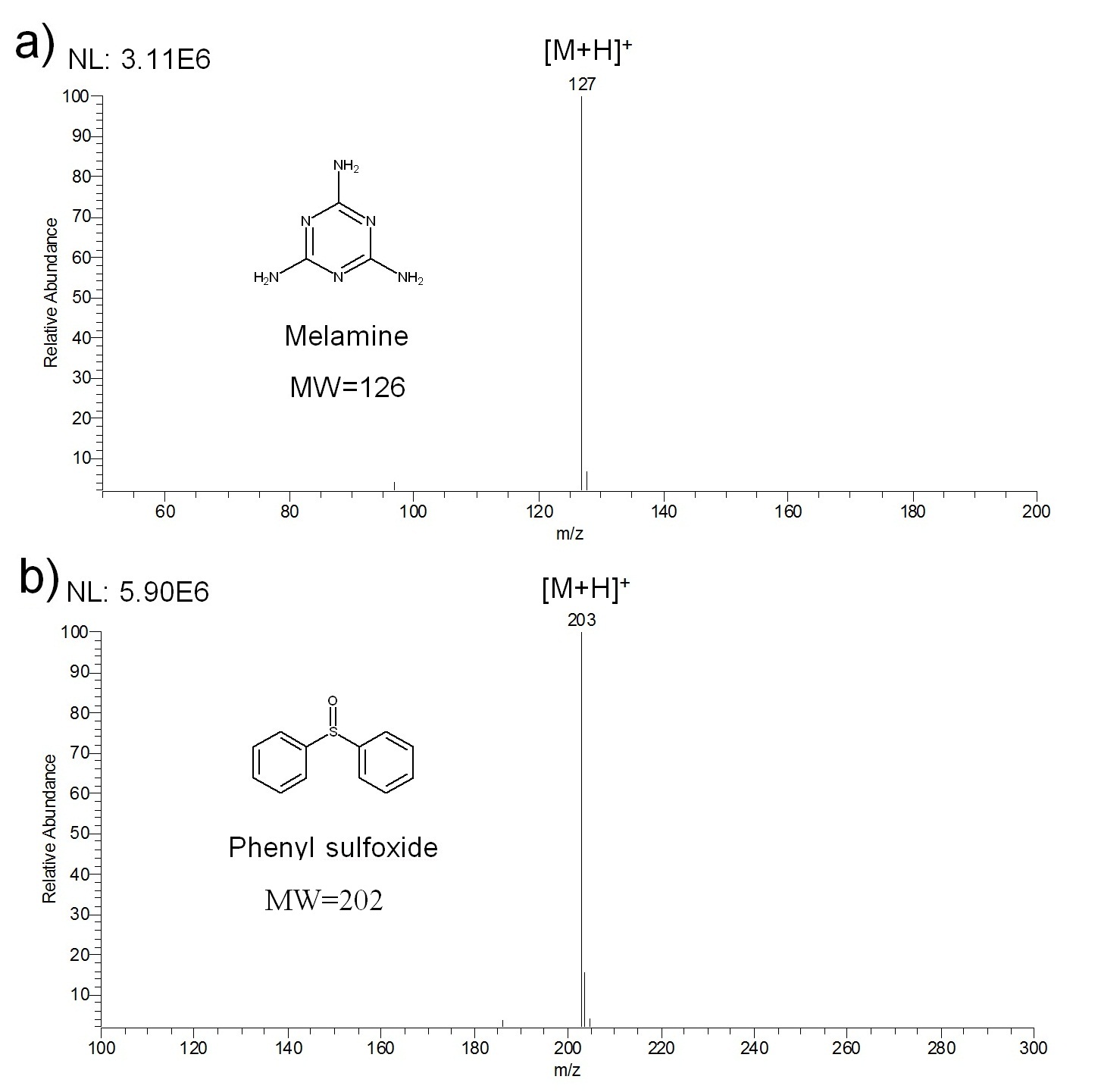
**

**Supplementary Figure S15**：The AFI mass spectra obtained with triple-quadrupole mass spectrometer: a) melamine, b) phenyl sulfoxide. The compounds were dissolved in the mixed solvents (V_H2O_:V_CH3CN_=1:1). The concentrations of compounds were 0.1 mg mL^-1^. The sample rob dipped approximately 0.5uL solution of compounds were directly subjected to the outer flame.

1. **Table**

Accurate mass identification results as listed in Supplementary Tablet S7.

**Supplementary Tablet S7**. Accurate mass identification for the major ionic species by AFI-MS.

| Compound | Precursor ions | | Ion elemental compositions | Detected *m/z* | | Calculated *m/z* | | Relative error (ppm) | |
| --- | --- | --- | --- | --- | --- | --- | --- | --- | --- |
| Methyl salicylate | [M+H]^+^ | | C_8_H_9_O_3_^+^ | 153.0546 | | 153.0546 | | 0.0 | |
| Dimethyl sulfoxide | [M+H]^+^ | | C_4_H_13_O_2_S_2_^+^ | 157.0352 | | 157.0351 | | 0.6 | |
| 2-Methoxyethyl ether | [M+H]^+^ | | C_6_H_15_O_3_^+^ | 135.1015 | | 135.1016 | | -0.7 | |
| Phenyl sulfoxide | [M+H]^+^ | | C_12_H_11_OS^+^ | 203.0524 | | 203.0525 | | -0.5 | |
| H-PHE-PHE-OH | [M+H]^+^ | | C_18_H_21_N_2_O_3_^+^ | 313.1546 | | 313.1547 | | -0.3 | |
| H-ALA-GLY-OH | [M+H]^+^ | | C_5_H_11_N_2_O_3_^+^ | 147.0764 | | 147.0764 | | 0.0 | |
| 5，6-Dimethylbenzimidazole | [M+H]^+^ | | C_9_H_11_N_2_^+^ | 147.0918 | | 147.0917 | | 0.7 | |
| 6-Chloroguanine | [M+H]^+^ | | C_5_H_5_ClN_5_^+^ | 170.0228 | | 170.0228 | | 0.0 | |
| Sudan1 | [M+H]^+^ | | C_16_H_13_N_2_O^+^ | 249.1021 | | 249.1022 | | -0.4 | |
| Anthracene | M^+^· | | C_14_H_10_^.+^ | 178.0777 | | 178.0777 | | 0.0 | |
| Propyphenazone | [M+H]^+^ | | C_14_H_19_N_2_O^+^ | 231.1492 | | 231.1492 | | 0.0 | |
| Azithromycin | [M+H]^+^ | | C_38_H_73_N_2_O_12_^+^ | 749.5156 | | 749.5158 | | -0.3 | |
| Metronidazole | [M+H]^+^ | | C_6_H_10_N_3_O_3_^+^ | 172.0716 | | 172.0717 | | -0.6 | |
| 4-Acetamidophenol | [M+H]^+^ | | C_8_H_10_NO_2_^+^ | 152.0706 | | 152.0706 | | 0.0 | |
| Pseudoephedrine | [M+H]^+^ | | C_10_H_16_NO^+^ | 166.1226 | | 166.1226 | | 0.0 | |
| Diphenhydramine | [M+H]^+^ | | C_17_H_22_NO^+^ | 256.1697 | | 256.1696 | | 0.4 | |
| Dextromethorphan | [M+H]^+^ | | C_18_H_26_NO^+^ | 272.2010 | | 272.2009 | | 0.4 | |
| Caffeine | [M+H]^+^ | | C_8_H_11_N4O_2_^+^ | 195.0876 | | 195.0877 | | -0.5 | |
| Allicin | [M+H]^+^ | | C_6_H_11_OS_2_^+^ | 163.0246 | | 163.0246 | | 0.0 | |
|  | [M+NH_4_]^+^ | | C_6_H_14_NOS_2_^+^ | 180.0512 | | 180.0511 | | 0.6 | |
| 3-fluorobenzoic acid | [M-H]^-^ | | C_7_H_4_FO_2_^-^ | 139.0200 | | 139.0201 | | -0.7 | |
| Ferrocene | M^+^· | | C_10_H_10_Fe^.+^ | 186.0126 | | 186.0126 | | 0.0 | |
| Imazalil | [M+H]^+^ | | C_14_H_15_Cl_2_N_2_O^+^ | 297.0557 | | 297.0556 | | 0.3 | |
|  | |  |  | | 299.0528 | | 299.0526 | | 0.7 |
